# Piezoresponse in Ferroelectric Materials under Uniform Electric Field of Electrodes

**DOI:** 10.3390/s21113707

**Published:** 2021-05-26

**Authors:** Artur Udalov, Denis Alikin, Andrei Kholkin

**Affiliations:** 1 School of Natural Sciences and Mathematics, Ural Federal University, 620000 Ekaterinburg, Russia; artur.udalov@urfu.ru (A.U.); kholkin@ua.pt (A.K.); 2 Department of Physics & CICECO—Aveiro Institute of Materials, University of Aveiro, 3810-193 Aveiro, Portugal

**Keywords:** piezoelectric response, capacitor geometry, piezoelectric materials, piezoresponse force microscopy, uniform electric field, interferometry, Doppler laser vibrometer, quantification

## Abstract

The analytical solution for the displacements of an anisotropic piezoelectric material in the uniform electric field is presented for practical use in the “global excitation mode” of piezoresponse force microscopy. The solution is given in the Wolfram Mathematica interactive program code, allowing the derivation of the expression of the piezoresponse both in cases of the anisotropic and isotropic elastic properties. The piezoresponse’s angular dependencies are analyzed using model lithium niobate and barium titanate single crystals as examples. The validity of the isotropic approximation is verified in comparison to the fully anisotropic solution. The approach developed in the paper is important for the quantitative measurements of the piezoelectric response in nanomaterials as well as for the development of novel piezoelectric materials for the sensors/actuators applications.

## 1. Introduction

Piezoelectric materials are an important class of materials with applications as sensors, actuators, resonators [1,2,3], electric energy harvesters [4,5], in various microelectronic devices [6,7,8], and in the piezoelectric catalysis for wastewater treatment [9]. Evaluating the effective piezoelectric coefficients via macroscopic response and predicting the response based on the known piezoelectric coefficients are vital in designing efficient electromechanical devices based on new materials. Piezoresponse force microscopy (PFM) is a local technique allowing the measurement of the surface piezoresponse with a unique high spatial resolution of 10–20 nm and vertical sensitivity around 100 fm [10,11]. PFM allows measuring the piezoresponse in the micro and nano-objects where macroscopic measurement techniques fail and PFM becomes the only way to quantify the material’s piezoelectric coefficients [12,13,14,15,16,17]. Despite the rapid development of the PFM technique within the last 30 years, many issues still remain, such as the contribution from the electrostatic force [18,19,20] and other parasitic effects [11,21,22,23], as well as the difficulties of the PFM response quantification, i.e., understanding the relationship between the measured surface displacement and components of the piezoelectric tensor [17,24,25,26,27,28].

The most straightforward PFM implementation is in the so-called “global excitation mode”, where an electric field is applied through micro- or nano-sized electrodes, while signal registration is performed utilizing scanning probe microscopy (SPM) probe rastering across the surface [16,29,30,31,32,33,34,35]. Global excitation can be realized via an electric field applied to the conductive tip across the tip-electrode interface and simply by the excitation of the electrode with probe acting as a mechanical sensor. In both cases, the solution of the problem of electromechanical response quantification in the applied electric field is close to the conventional laser interferometry problem of the response under the action of the uniform or close-to-the uniform electric field. Contrary to the highly non-uniform electric field from the SPM probe, where the solution is usually limited to the case of the uniform elastic properties or needs numerical calculations [24,26,27,36,37], the piezoelectric response in the uniform electric field allows an accurate solution. Lefki and Dormans derived the solution [38] for the case of the tetragonal (001) oriented ferroelectric materials bonded to the rigid substrate, as it is imperative to the interpretation of the Doppler vibrometer and laser interferometry data [39,40,41]. More complicated cases considering the flexible substrate contribution, top electrode lateral size, and multilayered structure were studied using sophisticated analytical models and finite element simulations [41,42,43,44,45,46,47].

In this paper, we provide the complete solution of this problem for the arbitrarily oriented ferroelectric sample covered by the top electrode and fixed on the rigid substrate for the cases of the transversally isotropic and fully anisotropic elastic properties. The solutions reveal the contribution of the elastic anisotropy to the piezoresponse, as well they are of interest for the determination of the piezoelectric coefficients from the displacement of the piezoelectric material in the uniform electric field. The results are important for understanding the “global excitation mode” of PFM and conventional PFM response in the case of the measurements in the piezoelectric thin films with the thickness much lower than the tip radius (e.g., for 2D piezoelectric materials) [48]. The solutions are represented in the Wolfram Mathematica interactive code, allowing the visualization and study of the equations in the crystals with arbitrary elastic/piezoelectric matrices and orientation.

## 2. Theoretical Framework

The direct piezoelectric effect is the appearance of the electric charge at the surface of the crystal with a specific symmetry class under the mechanical force’s action. The polarization vector is linearly connected with the mechanical stress components $\sigma_{kl}\;\left(k,l=1,2,3\right)$ by the equation:

(1) $$P_{i}=d_{ikl}\sigma_{kl},$$

where $d_{ikl}$ are the piezoelectric coefficients, representing the 3rd rank tensor.

PFM usually exploits the converse piezoelectric effect representing a change of the piezoelectric crystal dimensions under the external electric field’s action. The deformation tensor $\epsilon_{ij}$ is linearly dependent on the electric field vector $E_{i}$ in the mechanically free crystal ($\sigma_{ij}=0$):

(2) $$\epsilon_{ij}=d_{kij}E_{k}.$$

In both Equations (1) and (2), the same piezoelectric coefficient appears that can be justified by the thermodynamical analysis. In the general case, the third rank tensor has 27 independent components. $\epsilon_{ij}=\epsilon_{ji}$, $d_{kij}$ piezoelectric tensor is symmetrical by the two last indexes, which leads to the reduction of the independent component number to 18.

The problem of the crystal deformation under the action of the external electric field can be written in the isothermal state (θ=0) as:

$$\sigma_{ij}=c_{ijkl}^{E,T}\epsilon_{kl}-e_{ijm}^{T}E_{m},$$

(3) $$D_{m}=\varepsilon_{mk}^{\epsilon ,T}E_{k}+e_{mij}^{T}\epsilon_{ij},$$

where *D* is an electric induction, *T* is a temperature.

To shorten the notations, upper indexes will be further omitted. The equation of the motion for the piezoelectric media without an accounting of the mass forces can be written as:

(4) $$\frac{\partial \sigma_{ij}}{\partial x_{j}}=\rho \frac{\partial^{2}u_{i}}{\partial t^{2}}.$$

Neglecting the magnetic effects and in the quasi-static approximation for the electric field:

(5) $$\frac{\partial D_{i}}{\partial x_{i}}=0.$$

Substituting the expression for the electric field $E_{i}=-\frac{\partial \varphi }{\partial x_{i}}$ through the scalar potential to the equation of the state (3), the following can be obtained:

$$\sigma_{ij}=c_{ijkl}\frac{\partial u_{k}}{\partial x_{l}}+e_{kij}\frac{\partial \varphi }{\partial x_{k}}$$

(6) $$D_{i}=e_{ikl}\frac{\partial u_{k}}{\partial x_{l}}-\varepsilon_{ik}\frac{\partial \varphi }{\partial x_{k}}$$

The system of the linear equation of the piezoelectric media electro-elasticity can be obtained by the substitution equations of the state (6) to the equations of the motion (4) and electrostatics (5):

(7) $$c_{ijkl}\frac{\partial^{2}u_{k}}{\partial x_{l}\partial x_{j}}+e_{kij}\frac{\partial^{2}\varphi }{\partial x_{k}\partial x_{j}}=\rho \frac{\partial^{2}u_{i}}{\partial t^{2}},$$

(8) $$e_{ikl}\frac{\partial^{2}u_{k}}{\partial x_{l}\partial x_{i}}-\varepsilon_{ik}\frac{\partial \varphi }{\partial x_{k}\partial x_{i}}=0.$$

**Figure 1:**
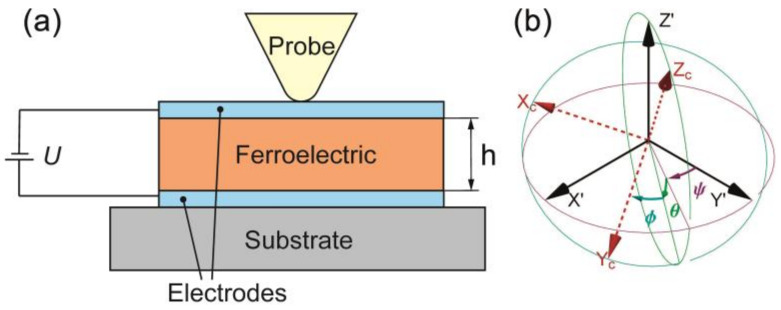
Schematics illustrating (**a**) geometry of the problem, (**b**) orientation of the axis, and corresponding choice of the Euler angles for the description of the material rotation.

Let us consider an infinite layer of the piezoelectric material with arbitrary anisotropy of the elastic, dielectric, and piezoelectric properties covered by the electrode from both sides and bonded on the rigid grounded substrate under constant voltage and in the condition of the free surface (Figure 1a). The task is one-dimensional, which means functions changing only along the axis z. In the equilibrium state, it transforms (7) to the system of the uniform differential equation, which can be expressed in the Voigt notation for the tensors as:

(9) $$\left\{\begin{array}{c} \begin{array}{c} e_{35}\varphi^{\prime \prime }+c_{55}u_{x}^{\prime \prime }+c_{45}u_{y}^{\prime \prime }+c_{35}u_{z}^{\prime \prime }=0\\ e_{34}\varphi^{\prime \prime }+c_{45}u_{x}^{\prime \prime }+c_{44}u_{y}^{\prime \prime }+c_{34}u_{z}^{\prime \prime }=0 \end{array}\\ \begin{array}{c} e_{33}\varphi^{\prime \prime }+c_{35}u_{x}^{\prime \prime }+c_{34}u_{y}^{\prime \prime }+c_{33}u_{z}^{\prime \prime }=0\\ e_{35}u_{x}^{\prime \prime }+e_{34}u_{y}^{\prime \prime }+e_{33}u_{z}^{\prime \prime }-\varepsilon_{33}\varphi^{\prime \prime }=0 \end{array} \end{array}\right.,$$

where *e_ij_* are piezoelectric constants.

The boundary conditions for the grounded stationary substrate:

(10) $$\left\{\begin{array}{c} {\left.\varphi \right|}_{z=0}=0\\ {\left.u_{x}\right|}_{z=0}=0\\ {\left.u_{y}\right|}_{z=0}=0\\ {\left.u_{z}\right|}_{z=0}=0 \end{array}\right..$$

The boundary conditions for the free electrode surface under the voltage U without applied force can be derived using the condition $\sigma_{ik}n_{k}=0$ [49] by the substitution $\sigma_{ik}$ from (6):

(11) $$\left\{\begin{array}{c} {\left.\varphi \right|}_{z=h}=U\\ {\left.\left(e_{35}\varphi^{\prime }+c_{55}u_{x}^{\prime }+c_{45}u_{y}^{\prime }+c_{35}u_{z}^{\prime }\right)\right|}_{z=h}=0\\ {\left.\left(e_{34}\varphi^{\prime }+c_{45}u_{x}^{\prime }+c_{44}u_{y}^{\prime }+c_{34}u_{z}^{\prime }\right)\right|}_{z=h}=0\\ {\left.\left(e_{33}\varphi^{\prime }+c_{35}u_{x}^{\prime }+c_{34}u_{y}^{\prime }+c_{33}u_{z}^{\prime }\right)\right|}_{z=h}=0 \end{array}\right..$$

The solution of the boundary problem (9)–(11) can be derived as:

(12) $$\left\{\begin{array}{c} \varphi \left[z\right]=\frac{Uz}{h}\\ u_{x}\left[z\right]=-\frac{c_{35}c_{44}e_{33}-c_{34}c_{45}e_{33}-c_{34}c_{35}e_{34}+c_{33}c_{45}e_{34}+c_{34}^{2}e_{35}-c_{33}c_{44}e_{35}}{\left(c_{35}^{2}c_{44}-2c_{34}c_{35}c_{45}+c_{33}c_{45}^{2}+c_{34}^{2}c_{55}-c_{33}c_{44}c_{55}\right)}\cdot \frac{Uz}{h}\\ u_{y}\left[z\right]=-\frac{-c_{35}c_{45}e_{33}+c_{34}c_{55}e_{33}+c_{35}^{2}e_{34}-c_{33}c_{55}e_{34}-c_{34}c_{35}e_{35}+c_{33}c_{45}e_{35}}{\left(c_{35}^{2}c_{44}-2c_{34}c_{35}c_{45}+c_{33}c_{45}^{2}+c_{34}^{2}c_{55}-c_{33}c_{44}c_{55}\right)}\cdot \frac{Uz}{h}\\ u_{z}\left[z\right]=-\frac{c_{45}^{2}e_{33}-c_{44}c_{55}e_{33}-c_{35}c_{45}e_{34}+c_{34}c_{55}e_{34}+c_{35}c_{44}e_{35}-c_{34}c_{45}e_{35}}{\left(c_{35}^{2}c_{44}-2c_{34}c_{35}c_{45}+c_{33}c_{45}^{2}+c_{34}^{2}c_{55}-c_{33}c_{44}c_{55}\right)}\cdot \frac{Uz}{h} \end{array}\right.$$

The complete solution is represented as a Wolfram Mathematica interactive program code (Appendix A) [50], which allows extracting a solution for any given orientation of the piezoelectric plate. In order to study the solution of the boundary problem (9)–(11) illustrating the behavior of the piezoelectric material in the uniform electric field, the solution was simplified for the case of the barium titanate and lithium niobate single crystals, which are model ferroelectric materials with the tabulated piezoelectric properties. These crystals possess different symmetries at room temperature: tetragonal and rhombohedral, respectively.

## 3. Results and Discussion

### 3.1. Barium Titanate: Material Tensors

Piezoelectric constant tensor $e_{ij}^{0}$ and piezoelectric coefficient tensor $d_{ij}^{0}$ can be written for the tetragonal barium titanate (*4 mm* point group) in the laboratory system of the coordinates as following [51]:

(13) $$e_{ij}^{0}=\left(\begin{array}{cc} \begin{array}{ccc} 0 & 0 & 0\\ 0 & 0 & 0\\ e_{31}^{0} & e_{31}^{0} & e_{33}^{0} \end{array} & \begin{array}{ccc} 0 & e_{15}^{0} & 0\\ e_{15}^{0} & 0 & 0\\ 0 & 0 & 0 \end{array} \end{array}\right)$$

(14) $$d_{ij}^{0}=\left(\begin{array}{cc} \begin{array}{ccc} 0 & 0 & 0\\ 0 & 0 & 0\\ d_{31}^{0} & d_{31}^{0} & d_{33}^{0} \end{array} & \begin{array}{ccc} 0 & d_{15}^{0} & 0\\ d_{15}^{0} & 0 & 0\\ 0 & 0 & 0 \end{array} \end{array}\right)$$

To write analytical equations for the displacement $\vec{u}$ through the piezoelectric coefficients, Equation (15) will be used for the relation between the tensors $e_{ijk}$ and $d_{ijk}$, which can lead to the following relationships in the laboratory system of the coordinates:

(15) $$e_{mij}^{0}=d_{mkl}^{0}c_{klij}^{0}.$$

The anisotropic elastic properties for the barium titanate were taken from [52], while isotropic elastic properties: Young modulus and Poisson ratio were calculated by the Voigt averaging of the elastic tensor [53]. Piezoelectric and elastic properties used for the calculations are presented in Appendix B.

### 3.2. Barium Titanate: Isotropic Elastic Properties

In the approximation of the isotropic elastic properties, an elastic tensor can be reduced to the:

(16) $$c_{ij}^{0}=c_{iso}=\left(\begin{array}{cc} \begin{array}{ccc} \lambda +2\mu & \lambda & \lambda \\ \lambda & \lambda +2\mu & \lambda \\ \lambda & \lambda & \lambda +2\mu \end{array} & \begin{array}{ccc} 0\;\;\;\;\;\;\;\;\;\; & \;0 & \;\;\;\;\;\;\;\;\;\;0\\ 0\;\;\;\;\;\;\;\;\;\; & \;0 & \;\;\;\;\;\;\;\;\;\;0\\ 0\;\;\;\;\;\;\;\;\;\; & \;0 & \;\;\;\;\;\;\;\;\;\;0 \end{array}\\ \begin{array}{ccc} 0\;\;\;\;\; & 0 & \;\;\;\;\;0\\ 0\;\;\;\;\; & 0 & \;\;\;\;\;0\\ 0\;\;\;\;\; & 0 & \;\;\;\;\;0 \end{array} & \begin{array}{ccc} \mu \;\;\;\;\;\;\;\;\;\; & \;0 & \;\;\;\;\;\;\;\;\;\;0\\ 0\;\;\;\;\;\;\;\;\;\; & \mu & \;\;\;\;\;\;\;\;\;\;0\\ 0\;\;\;\;\;\;\;\;\;\; & \;0 & \;\;\;\;\;\;\;\;\;\;\mu \end{array} \end{array}\right)$$

where λ, μ-Lame elastic modulus, which can be determined through Young’s modulus, Y, and Poisson coefficient, ν, as follows:

(17) $$\begin{array}{l} \lambda =\frac{\nu Y}{\left(1+\nu \right)\left(1-2\nu \right)}\\ \mu =\frac{Y}{2\left(1+\nu \right)} \end{array}$$

For the description of the sample rotation in relation to the laboratory coordinate systems, Euler angles $\left\{\phi ,\theta ,\psi \right\}$ are used, which correspond to the subsequent counterclockwise rotation around the axis $Z_{1}X_{2}Z_{3}$ (Figure 1b). The rotational matrix used in the calculation can be found in Appendix C. The angle ϕ is responsible for the rotation of the crystal in relation to the crystallographic axis $Z_{c}$, angle θ-for the tilt of the axis $Z_{c}$ in relation to the axis Z, angle ψ-for the rotation of the plate with regard to the axis Z.

Equation (12) for the $\vec{u}$ displacement vector components can be written for the plate of the barium titanate with the thickness, h, infinite in the XY plane, and rotated at the random angles $\left\{\phi ,\theta ,\psi \right\}$ in the approximation of the elastic properties’ isotropy (16) as follows:

(18) $$\left\{\begin{array}{c} u_{x}\left[z,\phi ,\theta ,\psi \right]=\frac{\mathrm{Sin}\left[\theta \right]\mathrm{Sin}\left[\psi \right]2\left(1+\nu \right)\left(e_{31}^{0}-e_{33}^{0}+\left(2e_{15}^{0}+e_{31}^{0}-e_{33}^{0}\right)\mathrm{Cos}\left[2\theta \right]\right)}{Y}\cdot \frac{Uz}{h}\\ u_{y}\left[z,\phi ,\theta ,\psi \right]=\frac{\mathrm{Sin}\left[\theta \right]Cos\left[\psi \right]2\left(1+\nu \right)\left(e_{31}^{0}-e_{33}^{0}+\left(2e_{15}^{0}+e_{31}^{0}-e_{33}^{0}\right)\mathrm{Cos}\left[2\theta \right]\right)}{Y}\cdot \frac{Uz}{h}\\ u_{z}\left[z,\phi ,\theta ,\psi \right]=\frac{\mathrm{Cos}\left[\theta \right]\left(1+\nu \right)\left(2\nu -1\right)\left(2e_{15}^{0}+e_{31}^{0}+e_{33}^{0}-\left(2e_{15}^{0}+e_{31}^{0}-e_{33}^{0}\right)\mathrm{Cos}\;\left[2\theta \right]\right)}{2Y\left(1-\nu \right)}\cdot \frac{Uz}{h} \end{array}\right.$$

The solution does not depend on the angle ϕ, which is due to the material isotropy in the plane of the crystallographic axis $X_{c}Y_{c}$. The absolute value of the lateral response ($\sqrt{u_{x}^{2}+u_{y}^{2}}$) does not depend on the angle of the plate rotation around the *Z*-axis (ψ angle).

For the case of the isotropic elastic material according to (15), non-zero components of the piezoelectric constant tensor in the laboratory coordinate system can be derived through the piezoelectric coefficients as follows:

(19) $$\begin{array}{l} e_{15}^{0}=\frac{d_{15}^{0}Y}{2+2\nu }\\ e_{31}^{0}=-\frac{Y\left(d_{31}^{0}+d_{33}^{0}\nu \right)}{-1+\nu +2\nu^{2}}\\ e_{33}^{0}=\frac{Y\left(d_{33}^{0}\left(-1+\nu \right)-2d_{31}^{0}\nu \right)}{-1+\nu +2\nu^{2}} \end{array}$$

Thus, solution (18) can be represented through the piezoelectric coefficients as:

(20) $$\left\{\begin{array}{c} u_{x}\left[z,\phi ,\theta ,\psi \right]=\mathrm{Sin}\left[\theta \right]\mathrm{Sin}\left[\psi \right]\left(d_{31}^{0}-d_{33}^{0}+\left(d_{15}^{0}+d_{31}^{0}-d_{33}^{0}\right)\mathrm{Cos}\left[2\theta \right]\right)\cdot \frac{Uz}{h}\\ u_{y}\left[z,\phi ,\theta ,\psi \right]=\mathrm{Sin}\left[\theta \right]\mathrm{Cos}\left[\psi \right]\left(d_{31}^{0}-d_{33}^{0}+\left(d_{15}^{0}+d_{31}^{0}-d_{33}^{0}\right)\mathrm{Cos}\left[2\theta \right]\right)\cdot \frac{Uz}{h}\\ u_{z}\left[z,\phi ,\theta ,\psi \right]=\mathrm{Cos}\left[\theta \right]\left(\frac{d_{15}^{0}+d_{31}^{0}+d_{33}^{0}-2\nu (d_{15}^{0}-d_{31}^{0})}{4\left(\nu -1\right)}\right.+\\ \left.+\frac{\left(d_{15}^{0}+d_{31}^{0}-d_{33}^{0}\right)\left(2\nu -1\right)\mathrm{Cos}\left[2\theta \right]}{2\left(\nu -1\right)}\right)\cdot \frac{Uz}{h} \end{array}\right.$$

It should be noted that the lateral response in the uniform electric field approximation in the form (20) does not depend on the material’s elastic properties and has the only contribution from the piezoelectric coefficients.

### 3.3. Barium Titanate: Anisotropic Elastic Properties

The tensor of the anisotropic elastic modules for the crystal with the 4 mm symmetry can be written in the laboratory coordinate system as:

(21) $$c_{ij}^{0}=c_{4mm}=\left(\begin{array}{cc} \begin{array}{ccc} c_{11}^{0} & c_{12}^{0} & c_{13}^{0}\\ c_{12}^{0} & c_{11}^{0} & c_{13}^{0}\\ c_{13}^{0} & c_{13}^{0} & c_{33}^{0} \end{array} & \begin{array}{ccc} 0\; & 0\; & 0\;\\ 0\; & 0\; & 0\;\\ 0\; & 0\; & 0\; \end{array}\\ \begin{array}{ccc} 0\; & 0 & \;0\\ 0\; & 0 & \;0\\ 0\; & 0 & \;0 \end{array} & \begin{array}{ccc} c_{44}^{0} & \;0 & \;0\\ 0 & c_{44}^{0} & \;0\\ 0 & \;0 & \frac{1}{2}\left(c_{11}^{0}-c_{12}^{0}\right)\; \end{array} \end{array}\right)$$

Equation (12) for the $\vec{u}$ displacement vector components can be written for the anisotropic barium titanate plate rotated at random angles $\left\{\phi ,\theta ,\psi \right\}$ as:

(22) $$\left\{\begin{array}{c} u_{x}\left[z,\phi ,\theta ,\psi \right]=\frac{\mathrm{Sin}\left[\theta \right]\left(\begin{array}{c} (3c_{33}^{0}\left(e_{15}^{0}+e_{31}^{0}\right)+\left(c_{13}^{0}+2c_{44}^{0}\right)\left(e_{31}^{0}-3e_{33}^{0}\right)-\\ -c_{11}^{0}\left(3e_{15}^{0}+e_{33}^{0}\right)+4(c_{11}^{0}e_{15}^{0}+c_{33}^{0}\left(e_{15}^{0}+e_{31}^{0}\right)-\\ -\left(c_{13}^{0}+2c_{44}^{0}\right)e_{33}^{0})\mathrm{Cos}\left[2\;\theta \right]-(-c_{33}^{0}\left(e_{15}^{0}+e_{31}^{0}\right)+\\ +c_{11}^{0}\left(e_{15}^{0}-e_{33}^{0}\right)+\left(c_{13}^{0}+2c_{44}^{0}\right)\left(e_{31}^{0}+e_{33}^{0}\right))\mathrm{Cos}\left[4\theta \right]) \end{array}\right)}{\left(\begin{array}{c} -{\left(c_{13}^{0}\right)}^{2}+c_{11}^{0}c_{33}^{0}-2c_{13}^{0}c_{44}^{0}+3\left(c_{11}^{0}+c_{33}^{0}\right)c_{44}^{0}+\\ +4\mathrm{Cos}\left[2\theta \right]\left(-c_{11}^{0}+c_{33}^{0}\right)c_{44}^{0}+\\ +\mathrm{Cos}\left[4\theta \right]\left({\left(c_{13}^{0}\right)}^{2}-c_{11}^{0}c_{33}^{0}+\left(c_{11}^{0}+2c_{13}^{0}+c_{33}^{0}\right)c_{44}^{0}\right) \end{array}\right)}\cdot \frac{\mathrm{Sin}\left[\psi \right]Uz}{4h}\\ u_{x}\left[z,\phi ,\theta ,\psi \right]=\frac{\mathrm{Sin}\left[\theta \right]\left(\begin{array}{c} 3c_{33}^{0}\left(e_{15}^{0}+e_{31}^{0}\right)+\left(c_{13}^{0}+2c_{44}^{0}\right)\left(e_{31}^{0}-3e_{33}^{0}\right)-\\ -c_{11}^{0}\left(3e_{15}^{0}+e_{33}^{0}\right)+4(c_{11}^{0}e_{15}^{0}+c_{33}^{0}\left(e_{15}^{0}+e_{31}^{0}\right)-\\ -\left(c_{13}^{0}+2c_{44}^{0}\right)e_{33}^{0})\mathrm{Cos}\left[2\theta \right]-(c_{33}^{0}\left(e_{15}^{0}+e_{31}^{0}\right)-\\ -c_{11}^{0}\left(e_{15}^{0}-e_{33}^{0}\right)-\left(c_{13}^{0}+2c_{44}^{0}\right)\left(e_{31}^{0}+e_{33}^{0}\right))\mathrm{Cos}\left[4\theta \right] \end{array}\right)}{\left(\begin{array}{c} -{\left(c_{13}^{0}\right)}^{2}+c_{11}^{0}c_{33}^{0}-2c_{13}^{0}c_{44}^{0}+3\left(c_{11}^{0}+c_{33}^{0}\right)c_{44}^{0}+\\ +4\mathrm{Cos}\left[2\theta \right]\left(-c_{11}^{0}+c_{33}^{0}\right)c_{44}^{0}+\\ +\mathrm{Cos}\left[4\theta \right]\left({\left(c_{13}^{0}\right)}^{2}-c_{11}^{0}c_{33}^{0}+\left(c_{11}^{0}+2c_{13}^{0}+c_{33}^{0}\right)c_{44}^{0}\right) \end{array}\right)}\cdot \frac{\mathrm{Cos}\left[\psi \right]Uz}{h}\\ u_{z}\left[z,\phi ,\theta ,\psi \right]=\frac{\left(\begin{array}{c} -c_{33}^{0}e_{15}^{0}-c_{33}^{0}e_{31}^{0}-2c_{44}^{0}e_{31}^{0}-2c_{44}^{0}e_{33}^{0}-\\ \left.-c_{11}^{0}\left(3e_{15}^{0}+e_{33}^{0}\right)\right)+c_{13}^{0}\left(4e_{15}^{0}+e_{31}^{0}+e_{33}^{0}\right)+\\ +4\left(c_{11}^{0}e_{15}^{0}-c_{13}^{0}e_{15}^{0}+c_{44}^{0}\left(e_{31}^{0}-e_{33}^{0}\right)\right)\mathrm{Cos}\left[2\theta \right]+\\ +(c_{33}^{0}\left(e_{15}^{0}+e_{31}^{0}\right)-c_{11}^{0}\left(e_{15}^{0}-e_{33}^{0}\right)-\\ -\left(c_{13}^{0}+2c_{44}^{0}\right)\left(e_{31}^{0}+e_{33}^{0}\right))\mathrm{Cos}\left[4\theta \right]) \end{array}\right)}{\left(\begin{array}{c} -{\left(c_{13}^{0}\right)}^{2}+c_{11}^{0}c_{33}^{0}-2c_{13}^{0}c_{44}^{0}+3\left(c_{11}^{0}+c_{33}^{0}\right)c_{44}^{0}+\\ +4\mathrm{Cos}\left[2\theta \right]\left(c_{33}^{0}-c_{11}^{0}\right)c_{44}^{0}+\\ +\mathrm{Cos}\left[4\theta \right]\left({\left(c_{13}^{0}\right)}^{2}-c_{11}^{0}c_{33}^{0}+\left(c_{11}^{0}+2c_{13}^{0}+c_{33}^{0}\right)c_{44}^{0}\right) \end{array}\right)}\cdot \mathrm{Cos}\left[\theta \right]\frac{Uz}{h} \end{array}\right.$$

The anisotropic case’s solution becomes significantly more complicated, which is sourced by the diversification of the elastic properties in different crystallographic directions. By analogy with Equation (19), piezoelectric modules relate to the piezoelectric coefficients (21) as:

(23) $$\begin{array}{l} e_{15}^{0}=d_{15}^{0}c_{44}^{0}\\ e_{31}^{0}=d_{33}^{0}c_{13}^{0}+d_{31}^{0}\left(c_{11}^{0}+c_{12}^{0}\right)\\ e_{33}^{0}=2d_{31}^{0}c_{13}^{0}+d_{33}^{0}c_{33}^{0} \end{array}$$

Substitution of the (23) to the displacement vector $\vec{u}$ (22) leads to a significant complication of the equations, making it challenging to represent and intuitively interpret it in a general form.

### 3.4. Analysis of the Solution for the Barium Titanate: Anisotropic Elastic Properties

To illustrate the behavior of the solutions (18) and (22), they were analyzed for three orientations (cuts) of barium titanate crystals: (001)_c_, (011)_c,_ and (100)_c_, which correspond to the (0, 0, 0), (180°, 45°,0), and (90°, 90°, 0) values of the Euler angles, respectively. The solutions (18), (20), (22), and (22) through (23) for the displacement vector of the differently oriented crystals (chosen angles θ, ψ=0, and ∀ϕ) are summarized in Table 1. Equation (22) is well-known as the Lefki and Dormans solution [38]. The expression has the most simple shape for the case of the crystal cut along the possible polarization directions, while it becomes much more complicated for the case of (011)_c_ crystal orientation, where a combination of the elastic modulus contributes.

**Figure 2:**
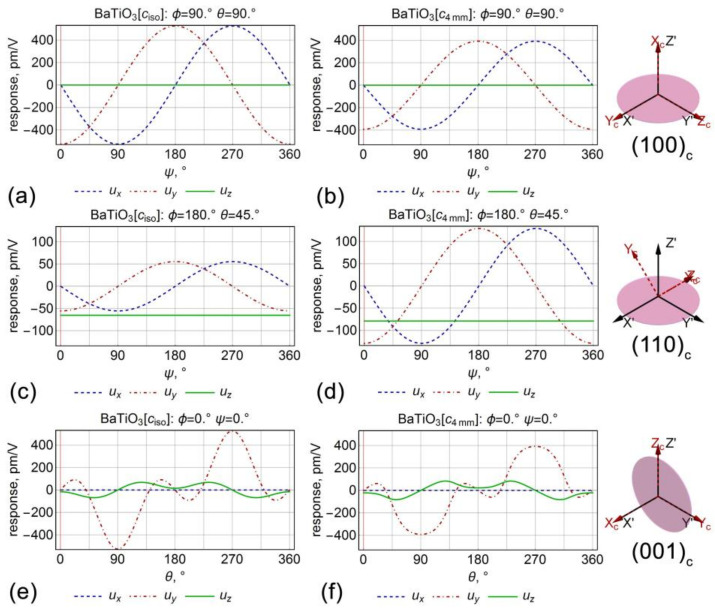
The dependencies of the displacement vector on the angles: (**a–d**) ψ and (**e**,**f**) θ in the barium titanate single crystal. (**a**,**c**,**e**) Isotropic and (**b**,**d**,**f**) anisotropic elastic properties.

Further, the response was analyzed in dependence on the angles θ and ψ (Figure 2), which was the equivalent in the “global excitation mode” PFM experiments to the choice of the specific cut of the crystal at the angle θ, or rotation of the sample under SPM probe at angle ψ, respectively. (100)_c_ cut of the crystal most effectively illustrates piezoresponse behavior with the crystal rotation (Figure 2a,b). The same as it is in the isotropic approximation, the rotation of the (100)_c_ oriented plate around the axis of the normal to the surface conserves the absolute value of the lateral piezoresponse, while $u_{x}$ and $u_{y}$ components transform by the cosine and sinus laws, respectively. It means that PFM in the “global excitation mode” should be gradually redistributed from the twisting to the buckling component of the cantilever motion [10]. As expected, normal piezoresponse, i.e., vertical PFM signal, was absent on this crystal cut. Comparison between anisotropic and isotropic cases revealed that the piezoresponse in the anisotropic case was around 20 percent lower. However, the general trend of piezoresponse behavior conserves for both cases (Figure 2a,b).

As expected, the normal piezoresponse in (001)_c_ had a contribution not only from the *d_33_* coefficient but from the *d_31_* as well (Table 1). In the case of the anisotropic elastic properties, contribution to the displacement vector from the d_31_ coefficient was proportional to the ratio between *c_33_* and *c_13_* elastic coefficients, which was about 0.92. In contrast, in the isotropic case, the displacement vector was proportional to the *ν/(ν − 1)*, which was about 0.67. Thus, the deviation of the response due to d_31_ was more significant for the case of the anisotropic elastic properties.

The plot of the surface displacement $\vec{u}$ vector components for the (010)_c_ cut of the crystal (θ=45°) depending on the angle ψ is presented in Figure 2c,d. In this case, the lateral response conserves the behavior, and a normal response appears, which was independent of the rotational angle. On this cut, the piezoresponse for the anisotropic crystal properties was more than twice as larger as the response calculated in the isotropic approximation. Other informative plots represent the dependencies of the lateral and vertical surface displacements on the angle *θ* (the angle between the outer normal to the plate and the crystallographic axis $Z_{c}$), which revealed a significant difference of the dependencies of the vertical and lateral piezoresponses in the isotropic and anisotropic cases (Figure 2e,f). Notably, not only the value of the piezoresponse changes after the transition from the anisotropic case to isotropic approximation but also the profile of the piezoresponse signal changes significantly.

### 3.5. Lithium Niobate Material Tensors

Lithium niobate is a rhombohedral material at room temperature, which belongs to *3m* point group [54]. The tensor of piezoelectric constants $e_{ij}^{0}$ and coefficients $d_{ij}^{0}$ in the laboratory coordinate system has the form:

(24) $$e_{ij}^{0}=\left(\begin{array}{cc} \begin{array}{ccc} 0 & 0 & 0\\ -e_{22}^{0} & e_{22}^{0} & 0\\ e_{31}^{0} & e_{31}^{0} & e_{33}^{0} \end{array} & \begin{array}{ccc} 0 & e_{15}^{0} & -e_{22}^{0}\\ e_{15}^{0} & 0 & 0\\ 0 & 0 & 0 \end{array} \end{array}\right)$$

(25) $$d_{ij}^{0}=\left(\begin{array}{cc} \begin{array}{ccc} 0 & 0 & 0\\ -d_{22}^{0} & d_{22}^{0} & 0\\ d_{31}^{0} & d_{31}^{0} & d_{33}^{0} \end{array} & \begin{array}{ccc} 0 & d_{15}^{0} & -d_{22}^{0}\\ d_{15}^{0} & 0 & 0\\ 0 & 0 & 0 \end{array} \end{array}\right)$$

The anisotropic elastic properties for the lithium niobate were taken from [54], while isotropic elastic properties, Young’s modulus and Poisson ratio, were calculated using Voigt averaging the elastic tensor [53]. Piezoelectric and elastic properties used for the calculations are presented in Appendix B.

### 3.6. Lithium Niobate: Isotropic Elastic Properties

The displacement vector $\vec{u}$ for XY-infinite lithium niobate plate of the thickness *h* rotated at arbitrary Euler angles $\left\{\phi ,\theta ,\psi \right\}$ can be written in the approximation of elastic properties isotropy (50) as:

(26) $$\left\{\begin{array}{c} u_{x}\left[z,\phi ,\theta ,\psi \right]=\left(1+\nu \right)\mathrm{Sin}\left[\theta \right](-\mathrm{Sin}\left[\theta \right](\mathrm{Cos}\left[\psi \right]\left(\mathrm{Sin}\left[\phi \right]+3\mathrm{Sin}\left[3\phi \right]\right)+\mathrm{Cos}\left[\theta \right](\mathrm{Cos}\left[\phi \right]+\\ +3\mathrm{Cos}\left[3\phi \right])\mathrm{Sin}\left[\psi \right])e_{22}^{0}+2\mathrm{Sin}\left[\psi \right]\left(e_{31}^{0}+\mathrm{Cos}\left[2\theta \right]\left(e_{15}^{0}+e_{31}^{0}-e_{33}^{0}\right)-e_{33}^{0}\right))\cdot \frac{Uz}{Yh}\\ u_{y}\left[z,\phi ,\theta ,\psi \right]=\mathrm{Cos}\left[\psi \right]\left(e_{31}^{0}-e_{33}^{0}+\left(2e_{15}^{0}+e_{31}^{0}-e_{33}^{0}\right)\mathrm{Cos}\left[2\theta \right]-e_{22}^{0}\mathrm{Cos}\left[3\phi ]\mathrm{Sin}[2\theta \right]\right)\;\;\\ \cdot \left(e_{22}^{0}\mathrm{Cos}\left[3\phi ]\mathrm{Sin}[2\theta \right]\right)+2e_{22}^{0}\mathrm{Sin}\left[\theta \right]\mathrm{Sin}\left[3\phi \right]\mathrm{Sin}\left[\psi \right])\frac{Uz\left(1+\nu \right)}{Yh}\\ u_{z}\left[z,\phi ,\theta ,\psi \right]=\left(1+\nu \right)\left(2\nu -1\right)(\left(-2e_{15}^{0}-e_{31}^{0}-e_{33}^{0}\right)\mathrm{Cos}\left[\theta \right]+\left(2e_{15}^{0}+e_{31}^{0}-e_{33}^{0}\right)\mathrm{Cos}\left[\theta \right]\mathrm{Cos}\left[2\theta \right]+\\ +2e_{22}^{0}\mathrm{Cos}\left[3\phi \right]\mathrm{Sin}{\left[\theta \right]}^{3})\cdot \frac{Uz}{2Yh\left(\nu -1\right)} \end{array}\right.$$

Similar to the case of the barium titanate, the absolute value of the lateral response ($\sqrt{u_{x}^{2}+u_{y}^{2}}$) does not depend on the angle of plate rotation around the *Z*-axis (angle ψ) but depends on the other angles. According to (15), the non-zero components of the piezoelectric constant tensor in the elastically isotropic material can be expressed in the laboratory coordinate system through the components of the elastic moduli tensor as follows:

(27) $$\begin{array}{l} \begin{array}{l} e_{15}^{0}=\frac{Yd_{15}^{0}}{2+2\nu }\\ e_{22}^{0}=\frac{Yd_{22}^{0}}{1+\nu }\;\; \end{array}\;\;\;\;\;\;\;\;\;\;\;\;\;\;\;\;\;\;\;\;\;\;\;\;\;\;\;\;\;\;\;\;\;\;\;\;\;\;\;\\ \begin{array}{l} e_{31}^{0}=-\frac{Y\left(d_{31}^{0}+\nu d_{33}^{0}\right)}{-1+\nu +2\nu^{2}}\;\;\;\;\;\;\;\;\;\;\;\;\;\;\;\;\;\;\;\\ e_{33}^{0}=\frac{Y\left(-2\nu d_{31}^{0}+\left(-1+\nu \right)d_{33}^{0}\right)}{-1+\nu +2\nu^{2}} \end{array} \end{array}$$

Therefore, the solution (26) is expressed through (27) as:

(28) $$\left\{\begin{array}{c} u_{x}\left[z,\phi ,\theta ,\psi \right]=-\mathrm{Sin}\left[\theta \right](\mathrm{Cos}\left[\psi \right]\mathrm{Sin}\left[\theta \right]\left(\mathrm{Sin}\left[\phi \right]+3\mathrm{Sin}\left[3\phi \right]\right)d_{22}^{0}+\mathrm{Sin}\left[\psi \right](\mathrm{Cos}\left[\theta \right]\cdot \;\\ \cdot \left(\mathrm{Cos}\left[\phi \right]+3\mathrm{Cos}\left[3\phi \right]\right)\mathrm{Sin}\left[\theta \right]d_{22}^{0}-2d_{31}^{0}-\mathrm{Cos}\left[2\theta \right]\left(d_{15}^{0}+2d_{31}^{0}-2d_{33}^{0}\right)+2d_{33}^{0}))\cdot \frac{Uz}{h}\\ u_{y}\left[z,\phi ,\theta ,\psi \right]=(\mathrm{Cos}\left[\psi \right]\left(d_{31}^{0}-d_{33}^{0}+\left(d_{15}^{0}+d_{31}^{0}-d_{33}^{0}\right)\mathrm{Cos}\left[2\theta \right]-d_{22}^{0}\mathrm{Cos}\left[3\phi ]\mathrm{Sin}[2\theta \right]\right)+\\ +2d_{22}^{0}\mathrm{Sin}\left[\theta \right]\mathrm{Sin}\left[3\phi \right]\mathrm{Sin}\left[\psi \right]))\cdot \frac{Uz}{2h}\\ u_{z}\left[z,\phi ,\theta ,\psi \right]=(\mathrm{Cos}\left[\theta \right]\left(d_{15}^{0}+d_{31}^{0}+d_{33}^{0}-2d_{15}^{0}\nu +2d_{31}^{0}\nu +\left(d_{15}^{0}+d_{31}^{0}-d_{33}^{0}\right)\left(-1+2\nu \right)\mathrm{Cos}\left[2\theta \right]\right)\\ +2d_{22}^{0}\left(-1+2\nu \right)\mathrm{Cos}\left[3\phi \right]\mathrm{Sin}{\left[\theta \right]}^{3})\frac{Uz}{2h\left(\nu -1\right)} \end{array}\right.$$

The lateral response in the form (28), as well as for barium titanate, does not depend on the elastic properties of the material.

### 3.7. Lithium Niobate: Anisotropic Elastic Properties

The tensor of elastic moduli for crystals of similar symmetry (3*m*) in the laboratory coordinate system:

(29) $$c_{ij}^{0}=c_{3m}^{0}=\left(\begin{array}{cc} \begin{array}{ccc} c_{11}^{0} & c_{12}^{0} & c_{13}^{0}\\ c_{12}^{0} & c_{11}^{0} & c_{13}^{0}\\ c_{13}^{0} & c_{13}^{0} & c_{33}^{0} \end{array} & \begin{array}{ccc} c_{14}^{0}\;\;\;\; & 0\;\;\;\;\;\;\;\;\;\;\;\;\; & 0\;\;\;\;\;\;\;\;\;\\ -c_{14}^{0}\;\;\;\; & 0\;\;\;\;\;\;\;\;\;\;\;\;\; & 0\;\;\;\;\;\;\;\;\;\\ 0\;\;\;\; & 0\;\;\;\;\;\;\;\;\;\;\;\;\; & 0\;\;\;\;\;\;\;\;\; \end{array}\\ \begin{array}{ccc} c_{14}^{0}\;\;\;\; & -c_{14}^{0} & \;\;\;0\\ 0\;\;\;\; & 0 & \;\;\;0\\ 0\;\;\;\; & 0 & \;\;\;0 \end{array} & \begin{array}{ccc} c_{44}^{0} & \;0 & \;0\\ 0 & c_{44}^{0} & \;c_{14}^{0}\\ 0 & \;c_{14}^{0} & \frac{1}{2}\left(c_{11}^{0}-c_{12}^{0}\right) \end{array} \end{array}\right)$$

The piezoelectric constants are expressed through the piezoelectric coefficients as:

(30) $$\begin{array}{l} \begin{array}{l} e_{15}^{0}=c_{44}^{0}d_{15}^{0}-c_{14}^{0}d_{22}^{0}\;\;\;\;\;\;\;\;\;\;\;\;\;\;\;\;\;\;\\ e_{22}^{0}=-c_{14}^{0}d_{15}^{0}+\left(c_{11}^{0}-c_{12}^{0}\right)d_{22}^{0} \end{array}\\ \begin{array}{l} e_{31}^{0}=c_{11}^{0}d_{31}^{0}+c_{12}^{0}d_{31}^{0}+c_{13}^{0}d_{33}^{0}\\ e_{33}^{0}=2c_{13}^{0}d_{31}^{0}+c_{33}^{0}d_{33}^{0}\;\;\;\;\;\;\;\;\;\;\;\;\;\;\; \end{array} \end{array}$$

The expression for the displacement vector $\vec{u}$ in the fully anisotropic case is too tedious to present here in the analytical form. Thereby, the solution is given in the main text only for some specific chosen Euler angles.

### 3.8. Analysis of the Solution for the Lithium Niobate: Anisotropic Elastic Properties

**Figure 3:**
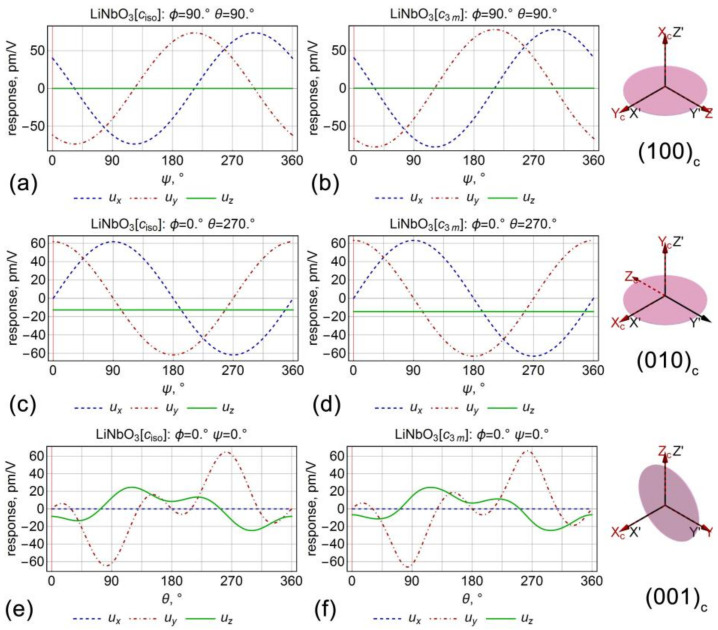
The dependencies of the displacement vector on the angles (**a**–**d**) ψ and (**e**,**f**) θ for the lithium niobate crystals. (**a**,**c**,**e**) Isotropic and (**b**,**d**,**f**) anisotropic elastic properties.

Analytical expressions for the displacement vector of points of the plate surface (z=h) at various angles θ (at ψ=0 and ∀ϕ) for the lithium niobate are summarized in Table 2. Analysis of the piezoresponse dependencies on the angle ψ revealed a similar trend as in barium titanate, but without such a large difference between isotropic and anisotropic cases. It did not exceed 3% for various orientations. It was also important that (100)_c_ and (010)_c_ oriented crystals possessed equivalent ψ-angle dependencies in the tetragonal barium titanate, while the dependencies were different for the lithium niobate, where piezoresponse did not nullify on (100)_c_ cut at the angle ψ=0 due to rhombohedral symmetry of the crystalline lattice (Figure 3a,b). The dependencies on the cut angle θ were also significantly different for the case of lithium niobate in comparison to barium titanate (Figure 2e,f and Figure 3e,f). In the lithium niobate case, a negligible difference between isotropic and anisotropic cases was observed, which demonstrated that the isotropic approximation’s applicability is highly dependent on the symmetry and elastic properties of the crystal.

Table 3 shows the ratios between the contributions to the piezoelectric response from each component of the piezoelectric coefficient tensor calculated for lithium niobate crystals with the different orientations. The total measured piezoresponse was taken as 100%. Negative values, thus, indicate that the given coefficient reduced the total piezoelectric response. For example, a major contribution to the piezoresponse in (001)_c_ cut lithium niobate comes from the d_33_ coefficient, while the contribution from the d_31_ coefficient reduces the total electromechanical response. d_15_ and d_22_ coefficients determine the response in the (100)_c_− and (010)_c_ nonpolar cuts. In contrast, the (011)_c_ cut of the crystal has a valuable contribution from all piezoelectric coefficients of the lithium niobate piezoelectric tensor, which makes difficult the recovery of piezoelectric coefficients in this orientation from the values of the measured piezoresponse.

## 4. Verification of the Analytical Model

### 4.1. Verification of the Analytical Model by the Finite Element Modeling (FEM)

The analytical results were verified by the quantitative comparison with the numerical solution of the same problem with FEM in the COMSOL Multiphysics software. The model was realized using “Electrostatics” and “Structural mechanics” modules. The piezoelectric sample of a finite-size was clamped to the rigid non-deformable steel plate. The constant potential was applied to the top surface, while the bottom surface of the piezoelectric plate was grounded. The rotation of the plate was performed with the same Euler matrix as used in the analytical theory (Appendix C). The comparison of the results was made for the (010)_c_ cut of the lithium niobate single crystal. The same properties of the crystal were used in the FEM and analytical model.

**Figure 4:**
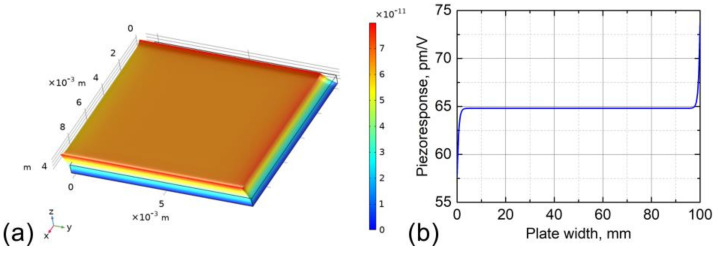
(**a**) Shear deformation of the (010)_c_ lithium niobate plate under the action of the uniform electric field. (**b**) The piezoresponse profile across the piezoelectric plate. The deformation of the plate is represented on the image 10^7^ times larger than the actual deformation shown at the color scale bar. Blackline contour shows an initial undeformed sample shape.

**Figure 5:**
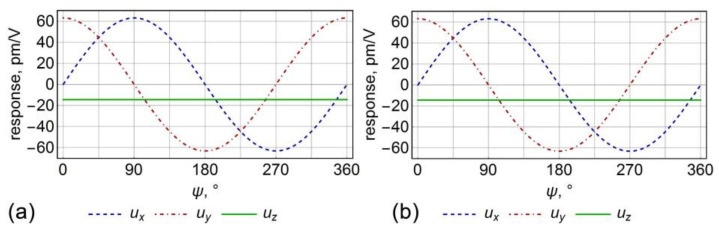
The dependencies of the displacement vector on the angles ψ for the (010)_c_ cut of the lithium niobate single crystals: (**a**) analytical theory and (**b**) finite element simulations.

The image illustrating the piezoelectric displacement in the electric field for the (010)_c_ cut of the lithium niobate single crystal is presented in Figure 4a. It is seen that shear deformation due to the *d_15_* shear coefficient dominates against other contributions. The top surface moved along x-direction in the sample Cartesian coordinate system (polarization direction in (010)_C_ crystal cut). At the same time, the bottom part of the plate was constrained to the substrate. The uniform electric field led to the uniform displacement of the piezoelectric surface almost in the whole area of the sample. The non-uniformity of the strain could be observed only immediately in the vicinity of the sample edges, which is related to the contribution of the sample edges in the fixed-size sample (enhanced electric field on the sample edges). This non-uniformity is not crucial if measurements are done far from the sample edges (Figure 4b). However, the electrode deposition in the experimental realization is also usually imperfect near the boundaries. Thus, the measurements near the sample edges should be avoided anyhow. Thereby, FEM verified well the validity of the one-dimensional approximation in the solution of the problem. The piezoresponse angular dependencies calculated with the analytical solution and FEM (data from the middle part of the sample) revealed perfect coincidence with each other (Figure 5).

### 4.2. Experimental Verification of the Analytical Model

**Figure 6:**
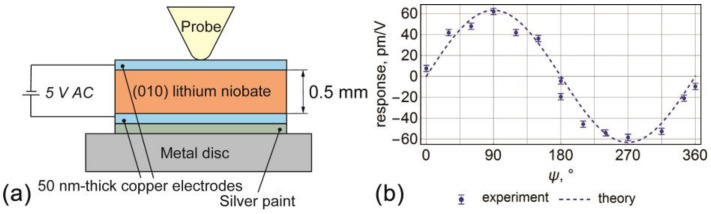
(**a**) Schematics of the “global-excitation” mode PFM measurements. (**b**) Theoretical and experimental dependencies of the lateral displacement on the angles ψ for the (010)_c_ cut of the lithium niobate single crystals.

Experimental verification of the theoretical results was performed in (010)_c_ cut crystal of the lithium niobate (Yamaju Ceramics, Owariasahi, Japan). 50-nanometer-thick copper electrodes were deposited on both crystal surfaces using magnetron sputtering. The sample was glued onto the metal disc by the silver paint (Figure 6a). “Global-excitation” mode PFM measurements were realized with the NTEGRA Aura scanning probe microscope (NT-MDT, Russia) using an external Zurich Instruments HF2LI lock-in amplifier. Commercial uncoated Multi−75EG (Budget Sensors, Bulgaria) cantilevers were utilized for the piezoresponse signal detection. The scheme illustrating experimental conditions is presented in Figure 6a. 5 V amplitude AC was applied to the bottom electrode while the top electrode and AFM tip were grounded. PFM measurements were done at low frequency (400 Hz) on the resonance-free plateau. The phase offset of the lock-in was kept at zero for all the measurements, and *X = Rcosφ* was measured in dependence on the rotational angle *ψ*. To calibrate vertical and lateral piezoresponse “inverted optical level sensitivity” calibration factors were extracted from the vertical and lateral force-distance curves’ measurements [55,56]. The values of the piezoresponse were divided by the amplitude of the applied AC voltage and by the respective vertical and lateral shape factors of the cantilever extracted using the procedure described in [56,57]. Lateral response angular dependence was measured and quantified (Figure 6b) to compare theoretical results with the experiments, while the vertical signal was captured in the 180-degree angular position, where the contribution of the cantilever buckling was minimal [22]. A pure vertical displacement was evaluated to be −14.5 pm/V. Both vertical and lateral measured responses matched well the results of the analytical model.

## 5. Conclusions

In this work, piezoelectric response in the uniform electric field created by the top electrode was analyzed for the case of the arbitrarily oriented piezoelectric material bonded to the rigid substrate, which presents “global excitation mode” PFM measurements. As the measurements of the piezoresponse angular dependencies often become crucial for interpreting the material piezoelectric properties [12], the particular focus in this paper was on the analysis of the angular dependencies. Based on the results of differently oriented barium titanate and lithium niobate crystals, the piezoelectric response was found to contain several contributions to the piezoelectric coefficients, which are often ignored in the measurements. The displacements were calculated for the case of fully coupled conditions both for the anisotropic and isotropic elastic properties. The anisotropic case was compared with the often considered transversally isotropic approximation. The difference between these two cases was shown to be significant for the case of barium titanate crystals and insignificant for lithium niobate. Thus, the isotropic approximation can be safely used only in specific crystals. The derived analytical theory was verified by finite element modeling and the global excitation mode PFM experiment demonstrated the very well coincidence.

Additionally, the solutions were represented in the Wolfram Mathematica interactive code, allowing easy access for researchers to the developed theoretical methodology. The code was shared in the Wolfram Notebook Archive cloud service and is available for the researcher community. The analytical model and interactive program code can be used to predict the piezoresponse angular behavior in various piezoelectric materials. This study is important for the further development of the quantitative “global excitation mode” PFM measurements, especially in low-dimensional materials, such as 2D and 1D piezoelectric nanostructures.

## Figures and Tables

**Table 1:** The expressions of the displacement vector for the case of the differently oriented crystals of the barium titanate derived from (18), (20), (22), (22) via (23).

θ $\vec{u}$	${\left(001\right)}_{c}$ $\left\{\begin{array}{c} u_{x}\\ u_{y}\\ u_{z} \end{array}\right\}$	${\left(100\right)}_{c}$ $\left\{\begin{array}{c} u_{x}\\ u_{y}\\ u_{z} \end{array}\right\}$	${\left(011\right)}_{c}$ $\left\{\begin{array}{c} u_{x}\\ u_{y}\\ u_{z} \end{array}\right\}$
	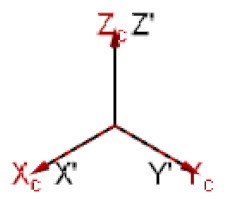	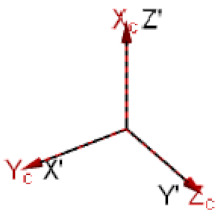	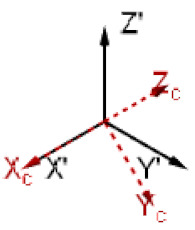
(18)	$\left\{\begin{array}{c} 0\\ 0\\ \frac{U\left(1+\nu \right)\left(1-2\nu \right)e_{33}^{0}}{Y\left(\nu -1\right)} \end{array}\right\}$	$\left\{\begin{array}{c} 0\\ \frac{2U\left(1+\nu \right)e_{15}^{0}}{-Y}\\ 0 \end{array}\right\}$	$\left\{\begin{array}{c} 0\\ \frac{U(\nu +1)(e_{31}^{0}\;-\;e_{33}^{0})}{\sqrt{2}Y}\\ \frac{U\left(1+\nu \right)\left(1-2\nu \right)\left(2e_{15}^{0}+e_{31}^{0}+e_{33}^{0}\right)}{2\sqrt{2}Y\left(\nu -1\right)} \end{array}\right\}$
(20)	$\left\{\begin{array}{c} 0\\ 0\\ \frac{2U\nu d_{31}^{0}}{\nu -1}-Ud_{33}^{0} \end{array}\right\}$	$\left\{\begin{array}{c} 0\\ -Ud_{15}^{0}\\ 0 \end{array}\right\}$	$\left\{\begin{array}{c} 0\\ \frac{\left(d_{31}^{0}-d_{33}^{0}\right)U}{\sqrt{2}}\\ \frac{U\left(\left(1-2\nu \right)d_{15}+\left(1+2\nu \right)d_{31}+d_{33}\right)}{2\sqrt{2}\left(\nu -1\right)} \end{array}\right\}$
(22)	$\left\{\begin{array}{c} 0\\ 0\\ -\frac{Ue_{33}^{0}}{c_{33}^{0}} \end{array}\right\}$	$\left\{\begin{array}{c} 0\\ -\frac{Ue_{15}^{0}}{c_{44}^{0}}\\ 0 \end{array}\right\}$	$\left\{\begin{array}{c} \frac{\begin{array}{c} -U\left(\left(c_{13}^{0}+2c_{44}^{0}\right)\left(e_{31}^{0}-e_{33}^{0}\right)-c_{33}^{0}\left(e_{15}^{0}\left.-e_{31}^{0}\right)+\right.\right.\\ \left.+c_{11}^{0}\left(e_{15}^{0}+e_{33}^{0}\right)\right) \end{array}}{\sqrt{2}\left({\left(c_{13}^{0}\right)}^{2}+2c_{13}^{0}c_{44}^{0}-c_{33}^{0}c_{44}^{0}-c_{11}^{0}\left(c_{33}^{0}+c_{44}^{0}\right)\right)}\\ \frac{\begin{array}{c} U\left(c_{33}^{0}\left(e_{15}^{0}+e_{31}^{0}\right)\right.-\\ \left.\left.-c_{13}^{0}\left(2e_{15}^{0}\right.+e_{31}^{0}+e_{33}^{0}\right)+c_{11}^{0}\left(e_{15}^{0}+e_{33}^{0}\right)\right) \end{array}}{\sqrt{2}\left({\left(c_{13}^{0}\right)}^{2}+2c_{13}^{0}c_{44}^{0}-c_{33}^{0}c_{44}^{0}-c_{11}^{0}\left(c_{33}^{0}+c_{44}^{0}\right)\right)} \end{array}\right\}$
(22)	$\left\{\begin{array}{c} 0\\ 0\\ -\frac{2c_{13}^{0}Ud_{31}^{0}}{c_{33}^{0}}-Ud_{33}^{0} \end{array}\right\}$	$\left\{\begin{array}{c} 0\\ -Ud_{15}^{0}\\ 0 \end{array}\right\}$	$\left\{\begin{array}{c} \frac{\begin{array}{c} -U\left(-{\left(c_{33}^{0}\right)}^{2}d_{33}^{0}+4\left(c_{13}^{0}+2c_{44}^{0}\right)\left(c_{12}^{0}d_{31}^{0}-\right.\right.\\ \left.-2c_{13}^{0}d_{31}^{0}+c_{13}^{0}d_{33}^{0}\right)+c_{11}^{0}\left(-c_{44}^{0}\left(d_{15}^{0}-8d_{31}^{0}\right)+\right.\\ \left.+\left(c_{12}^{0}-2c_{13}^{0}+3c_{33}^{0}\right)d_{31}^{0}+\left(c_{13}^{0}-3c_{33}^{0}\right)d_{33}^{0}\right)+\\ +c_{33}^{0}\left(c_{44}^{0}d_{15}^{0}+3c_{12}^{0}d_{31}^{0}-d_{33}^{0}\left(c_{13}^{0}+8c_{44}^{0}\right)-\right.\\ \left.\left.-2c_{13}^{0}d_{31}^{0}\right)+{\left(c_{11}^{0}\right)}^{2}d_{31}^{0}\right) \end{array}}{2\sqrt{2}\left({\left(c_{13}^{0}\right)}^{2}+2c_{13}^{0}c_{44}^{0}-c_{33}^{0}c_{44}^{0}-c_{11}^{0}\left(c_{33}^{0}+c_{44}^{0}\right)\right)}\\ \frac{\begin{array}{c} U\left(c_{13}^{0}\left(2c_{44}^{0}d_{15}^{0}\right.\right.\left.+c_{12}^{0}d_{31}^{0}\right)+\\ +{\left(c_{13}^{0}\right)}^{2}\left(2d_{31}^{0}+d_{33}^{0}\right)+c_{33}^{0}\left(c_{44}^{0}d_{15}^{0}+c_{12}^{0}d_{31}^{0}\right)+\\ +c_{11}^{0}\left(c_{44}^{0}d_{15}^{0}+\left(c_{13}^{0}+c_{33}^{0}\right)d_{31}^{0}+c_{33}^{0}d_{33}^{0}\right.) \end{array}}{\sqrt{2}\left({\left(c_{13}^{0}\right)}^{2}+2c_{13}^{0}c_{44}^{0}-c_{33}^{0}c_{44}^{0}-c_{11}^{0}\left(c_{33}^{0}+c_{44}^{0}\right)\right)} \end{array}\right\}$

**Table 2:** The expressions of the displacement vector for the case of the differently oriented crystals of the lithium niobate derived from (26), (27), (12) via (29), (12) via (29), and (30).

θ .. $\vec{u}$	${\left(001\right)}_{c}$ $\left\{\begin{array}{c} u_{x}\\ u_{y}\\ u_{z} \end{array}\right\}$	${\left(100\right)}_{c}$ $\left\{\begin{array}{c} u_{x}\\ u_{y}\\ u_{z} \end{array}\right\}$
(26)	$\left\{\begin{array}{c} 0\\ 0\\ \frac{\left(1+\nu \right)\left(1-2\nu \right)e_{33}^{0}U}{Y\left(\nu -1\right)} \end{array}\right\}$	$\left\{\begin{array}{c} \frac{2e_{22}^{0}U\left(1+\nu \right)}{Y}\\ -\frac{2e_{15}^{0}U\left(1+\nu \right)}{Y}\\ 0 \end{array}\right\}$
(27)	$\left\{\begin{array}{c} 0\\ 0\\ \frac{2U\nu d_{31}^{0}}{\nu -1}-Ud_{33}^{0} \end{array}\right\}$	$\left\{\begin{array}{c} 2d_{22}^{0}U\\ -d_{15}^{0}U\\ 0 \end{array}\right\}$
(12) via (29)	$\left\{\begin{array}{c} 0\\ 0\\ -\frac{Ue_{33}^{0}}{c_{33}^{0}} \end{array}\right\}$	$\left\{\begin{array}{c} -\frac{2\left(c_{14}^{0}e_{15}^{0}+c_{44}^{0}e_{22}^{0}\right)U}{2c_{14}^{0}{}^{2}+\left(c_{12}^{0}-c_{11}^{0}\right)c_{44}^{0}}\\ \frac{\left(c_{11}^{0}e_{15}^{0}-c_{12}^{0}e_{15}^{0}+2c_{14}^{0}e_{22}^{0}\right)U}{2c_{14}^{0}{}^{2}+\left(c_{12}^{0}-c_{11}^{0}\right)c_{44}^{0}}\\ 0 \end{array}\right\}$
(12) via (29) and (30)	$\left\{\begin{array}{c} 0\\ 0\\ -\frac{2c_{13}^{0}Ud_{31}^{0}}{c_{33}^{0}}-Ud_{33}^{0} \end{array}\right\}$	$\left\{\begin{array}{c} \frac{2\left(c_{14}^{0}{}^{2}+\left(-c_{11}^{0}+c_{12}^{0}\right)c_{44}^{0}\right)d_{22}^{0}U}{2c_{14}^{0}{}^{2}+\left(c_{12}^{0}-c_{11}^{0}\right)c_{44}^{0}}\\ \frac{\left(-2c_{14}^{0}{}^{2}d_{15}^{0}+\left(c_{11}^{0}-c_{12}^{0}\right)c_{44}^{0}d_{15}^{0}+\left(c_{11}^{0}-c_{12}^{0}\right)c_{14}^{0}d_{22}^{0}\right)U}{2c_{14}^{0}{}^{2}+\left(c_{12}^{0}-c_{11}^{0}\right)c_{44}^{0}}\\ 0 \end{array}\right\}$

**Table 3:** The ratios of the contributions to the piezoresponse in the lithium niobate from each of the piezoelectric tensor components.

	**(001)_c_**				**(011)_c_**			
	*d_15_*	*d_22_*	*d_31_*	*d_33_*	*d_15_*	*d_22_*	*d_31_*	*d_33_*
*u_x_*	0	0	0	0	0	0	0	0
*u_y_*	0	0	0	0	0	157%	−4%	−53%
*u_z_*	0	0	−5%	105%	60%	26%	−2%	16%
	**(010)_c_**				**(100)_c_**			
	*d_15_*	*d_22_*	*d_31_*	*d_33_*	*d_15_*	*d_22_*	*d_31_*	*d_33_*
*u_x_*	0	0	0	0	0	100%	0	0
*u_y_*	101%	−1%	0	0	96%	4%	0	0
*u_z_*	0	100%	0	0	0	0	0	0

**Figure A1:**
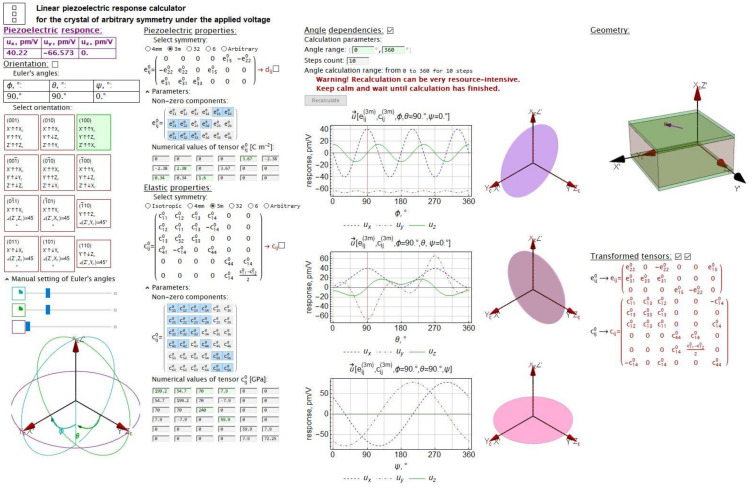
The interface of the developed interactive program code in the Wolfram Mathematica allowing analysis of the piezoresponse angular dependencies in the arbitrary oriented piezoelectric material. The program code can be found in [50].

## Data Availability

Data is contained within the article.

## Appendix B. Piezoelectric and Elastic Coefficients Used for the Calculations

**Table A1:** Piezoelectric and elastic coefficients for the barium titanate from [52] used for the calculations in the paper. Young modulus and Poisson coefficient were calculated by the Voigt averaging the elastic tensor [53]. Adapted from Li, Z.; Chan et al. The elastic and electromechanical properties of tetragonal BaTiO_3_ single crystals. *J. Appl. Phys.* **1991**, *70*, 7327–7332, with the permission of AIP Publishing.

$e_{15}^{0}=21.3\frac{C}{m^{2}}$	$e_{31}^{0}=-2.69\frac{C}{m^{2}}$	$e_{33}^{0}=3.65\frac{C}{m^{2}}$
$c_{11}^{0}=275\;\mathrm{GPA}$	$c_{12}^{0}=179\;\mathrm{GPA}$	$c_{13}^{0}=152\;\mathrm{GPA}$
$c_{33}^{0}=165\;\mathrm{GPA}$	$c_{44}^{0}=54.3\;\mathrm{GPA}$	
*Y* = 113 GPa	*ν* = 0.4	

**Table A2:** Piezoelectric and elastic coefficients for the lithium niobate from [54] used for the calculations in the paper. Young modulus and Poisson coefficient were calculated by Voigt averaging of the elastic tensor [53]. Adapted from Andrushchak, A.S. et al. Complete sets of elastic constants and photo elastic coefficients of pure and MgO-doped lithium niobate crystals at room temperature. *J. Appl. Phys.* **2009**, *106*, 073510, with the permission of AIP Publishing.

$e_{15}^{0}=3.67\frac{C}{m^{2}}$	$e_{22}^{0}=2.38\frac{C}{m^{2}}$	$e_{31}^{0}=0.34\frac{C}{m^{2}}$
$e_{33}^{0}=1.6\frac{C}{m^{2}}$	$c_{11}^{0}=199.2\;\mathrm{GPA}$	$c_{12}^{0}=54.7\;\mathrm{GPA}$
$c_{13}^{0}=70\;\mathrm{GPA}$	$c_{14}^{0}=7.9\;\mathrm{GPA}$	$c_{33}^{0}=240\;\mathrm{GPA}$
$c_{44}^{0}=59.9\;\mathrm{GPA}$		
*Y* = 170 GPa	ν = 0.25	

## Appendix C. The Rotation Matrix Used in the Calculations and Simulations

$$Z_{1}X_{2}Z_{3}=\left(-\begin{array}{ccc} \mathrm{Cos}\left[\phi \right]\mathrm{Cos}\left[\psi \right]-\mathrm{Cos}\left[\theta \right]\mathrm{Sin}\left[\phi \right]\mathrm{Sin}\left[\psi \right] & \mathrm{Cos}\left[\psi \right]\mathrm{Sin}\left[\phi \right]+\mathrm{Cos}\left[\theta \right]\mathrm{Cos}\left[\phi \right]\mathrm{Sin}\left[\psi \right] & \mathrm{Sin}\left[\theta \right]\mathrm{Sin}\left[\psi \right]\\ \mathrm{Cos}\left[\phi \right]\mathrm{Cos}\left[\psi \right]\mathrm{Sin}\left[\phi \right]-\mathrm{Cos}\left[\phi \right]\mathrm{Sin}\left[\psi \right] & \mathrm{Cos}\left[\theta \right]\mathrm{Cos}\left[\phi \right]\mathrm{Cos}\left[\psi \right]-\mathrm{Sin}\left[\phi \right]\mathrm{Sin}\left[\psi \right] & \mathrm{Cos}\left[\psi \right]\mathrm{Sin}\left[\theta \right]\\ \mathrm{Sin}\left[\theta \right]\mathrm{Sin}\left[\phi \right] & -\mathrm{Cos}\left[\phi \right]\mathrm{Sin}\left[\theta \right] & \mathrm{Cos}\left[\theta \right] \end{array}\right)$$
